# Crouzon's Syndrome: A Case Report

**DOI:** 10.5005/jp-journals-10005-1183

**Published:** 2013-04-26

**Authors:** G Ravi Kumar, M Jyothsna, Syed Basheer Ahmed, K Sree Lakshmi

**Affiliations:** Associate Professor, Department of Pedodontics and Preventive Dentistry, Government Dental College and Hospital, Rajiv Gandhi Institute of Medical Sciences, Kadapa, Andhra Pradesh, India; Associate Professor, Department of Oral Pathology, Government Dental College and Hospital, Rajiv Gandhi Institute of Medical Sciences, Kadapa, Andhra Pradesh, India; Associate Professor, Department of Prosthodontics and Crown and Bridge, Government Dental College and Hospital, Rajiv Gandhi Institute of Medical Sciences, Kadapa, Andhra Pradesh, India; Tutor, Department of Pediatric Dentistry, Government Dental College and Hospital, Rajiv Gandhi Institute of Medical Sciences, Kadapa Andhra Pradesh, India

**Keywords:** Craniosynostosis, Hypertelorism, Exophthalmos, Midfacial hypoplasia

## Abstract

Crouzon's syndrome (CS) is a rare autosomal dominant condition with multiple mutations of the fibroblast growth factor receptor (FGFR2) gene, which accounts for 4.8% of all cases of craniosynostosis. It is characterized by premature closure of cranial sutures, cranial deformities, midface hypoplasia, relative mandibular prognathism, hypertelorism, proptosis, strabismus and short upper lip, crowding of teeth, pseudocleft or sometimes cleft palate and other associated abnormalities. The CS can vary in severity from mild presentation to severe forms involving multiple cranial sutures. We report a case of CS in 11-year-old boy.

**How to cite this article:** Kumar GR, Jyothsna M, Ahmed SB, Lakshmi KS, Crouzon's Syndrome: A Case Report. Int J Clin Pediatr Dent 2013;6(1):33-37.

## INTRODUCTION

Crouzon's syndrome (CS) was described as one of the varieties of craniosynostosis caused by premature obliteration and ossification of two or more sutures. In 1912 a French neurologist, Octave Crouzon (1874-1938) first described a hereditary syndrome of craniofacial dysostosis in a mother and her daughter which included a triad-cranial deformities, facial anomalies and exophthalmos.^1^ The genetic defect appears to emanate from the mutation of fibroblast growth factor receptor 2 (FGFR2) on chromosome locus 10q25-q26, resulting in early fusion of skull bones during fetal development.^2^ It may be transmitted as an autosomal dominant inheritance but 25% of cases represent a fresh mutations.^3^ It accounts for approximately 4.8% of all cases of craniosynostosis with the prevalence of approximately 1 per 25,000 live births worldwide and has no sex or race predilection.^4,5^

Over 100 syndromes with craniosynostoses have been described of which Apert and Crouzon's syndromes are well known. CS presents similar craniosynostosis as in the Apert, Pfeiffer and Saethre-Chotzen syndromes except with no digital abnormalities.^6^ The appearance of CS can vary in severity from a mild to severe forms with multiple fused cranial sutures and marked midface and ocular defects. Mental retardation is not a hallmark feature unless premature closure of the cranial suture lines impairs brain development.^7^ We report one such case of CS showing all the classical features.

## CASE REPORT

**Figs 1A and B:**
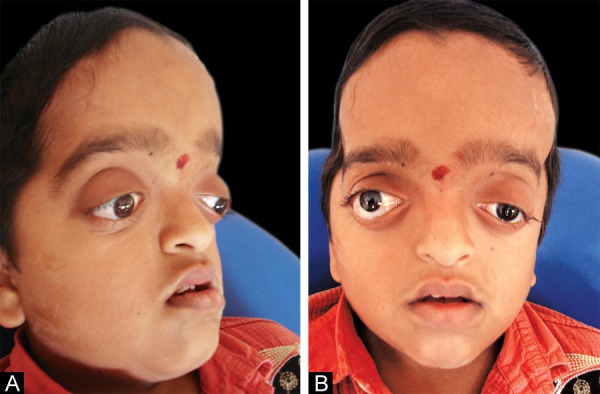
(A) Frontal and lateral view face showing the frontal bossing, midface hypoplasia and a relatively large mandible, shallow orbits, hypertelorism, exophthalmos, short and incompetent upper lip, depressed nasal bridge, (B) lateral view of the face

An 11-year-old boy reported to the Department of Pediatric Dentistry, Government Dental College and Hospital, Rajiv Gandhi Institute of Medical Sciences, Kadapa, India, with the chief complaint of irregularity in the arrangement of teeth. The patient presented with obvious dysmorphic cranial and facial features. On examination, enlarged cranial vault with frontal bossing, maxillary hypoplasia and a relative, mandibular prognathism was found. Ocular manifestations such as shallow orbits, hypertelorism, bilateral proptosis, exophthalmos and strabismus were present. Other facial features included short and incompetent upper lip, depressed nasal bridge and low-set ears but without any hearing loss (Figs 1A and B). His hands and feet found to be normal. Past medical history from the parents revealed that the features started developing slowly after birth and was diagnosed as CS at the age of 9 months and the patient had undergone cranial surgery to relieve closed sutures of the skull at the same age.

The prenatal, delivery and postnatal history was found to be insignificant. Family history revealed no abnormality. He is the first child of clinically healthy parents of nonconsanguineous marriage. His developmental milestones were found to be normal. The patient was presented with normal intelligence and little speech difficulty. At the time of his birth, his father was 36 years old and his mother was 22-year-old.

**Fig. 2:**
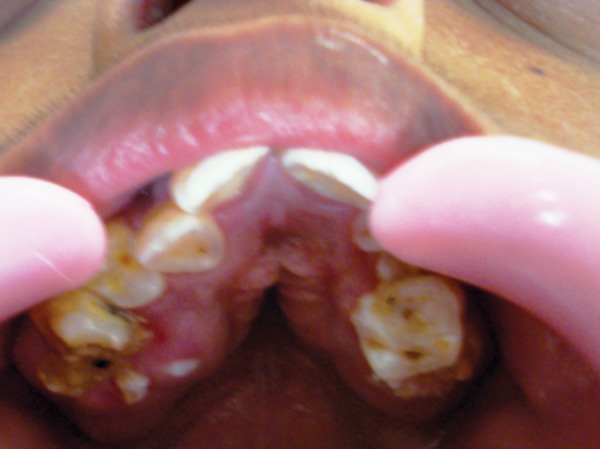
Pseudocleft in the palate

**Fig. 3:**
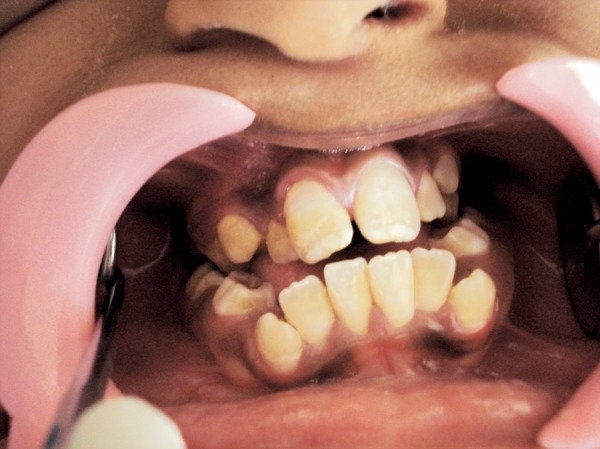
Intraoral view showing crowding of upper and lower teeth, reverse overjet with posterior crossbite and anterior open bite

**Fig. 4:**
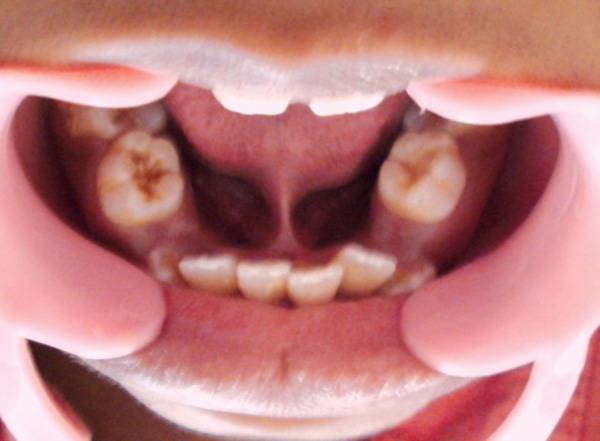
Tongue tie

On intraoral examination, V-shaped maxillary arch with high-arched palate and bilateral palatal swellings which was mimicking a pseudocleft (Fig. 2), retruded maxilla with a relatively large mandible were found. His oral hygiene was poor with crowding of upper and lower teeth, reverse over-jet with posterior crossbite and anterior open bite (Fig. 3), tongue tie (Fig. 4) and decayed teeth were present.

**Figs 5A and B:**
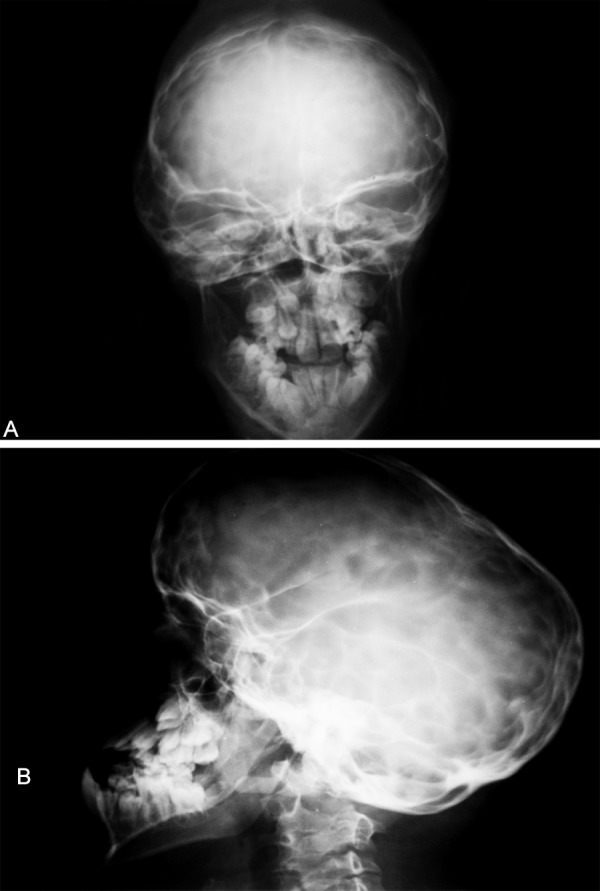
AP and lateral view of the skull–demonstrating maxillary retrusion, relative mandibular prognathism and ‘hammered silver’ (beaten metal/copper beaten) appearance

The skull radiographs revealed the ‘scaphocephalic’ skull shape, hypoplastic maxilla and zygoma with shallow orbits (Figs 5A and B). Prominent cranial markings of the inner surface of the cranial vault seen as multiple radiolucencies appearing as depressions resulting in the ‘hammered silver’ (beaten metal/copper beaten) appearance indicating internal remodeling of the calvaria due to an increase in intracranial pressure as a result of premature cranial suture fusion.

**Figs 6A to E:**
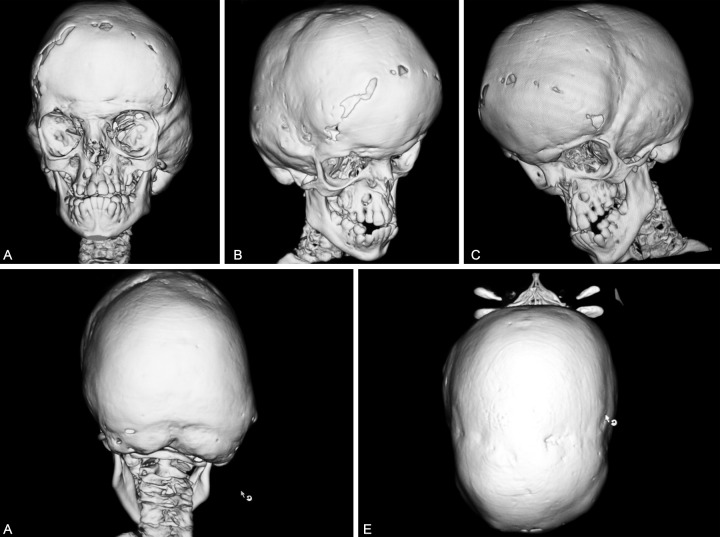
(A to C) Three-dimensional CT scan of skull revealing surgically opened coronal sutures, frontal bossing, shallow orbits, hypoplastic maxilla and zygoma; (D and E) obliteration of sagittal and lambdoid sutures

**Figs 7A and B:**
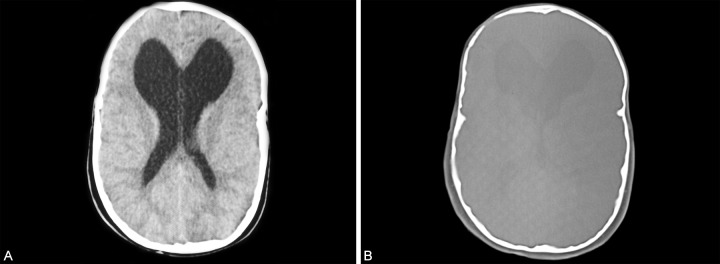
Moderate degree of hydrocephalus, diffuse indentation of inner table of skull seen in the CT scan

Three-dimensional computed tomographic (CT) scans of the skull showed fused sagittal and lambdoid sutures and surgically opened coronal sutures (Figs 6A to E), moderate degree of hydrocephalus with diffuse indentation of inner table of skull (Figs 7A and B). Other systemic examination was found to be normal. Routine hematological and biochemical tests were within normal limits.

## DISCUSSION

CS is inherited as an autosomal dominant fashion but there is an equal incidence of sporadic cases which probably represent new mutations. The sporadic cases are postulated to be associated with advanced paternal age and some investigators have found that this mutation is more common in the sperm of older men.^8^ However, the fact that the same mutation can produce a wide range of phenotypic expression makes the mechanism of anomalous development more complex.^9^ Penetrance is high although severity is variable. Within the family, members tend to have similar facial deformities but variable calvarial deformities and this phenotypic heterogeneity makes genetic counseling difficult.^9^ The phenotypic features of CS may be absent at birth and evolve gradually during the first few years of life.^10^The variability in both cranial and facial malformations depends on the order and rate of progression of sutural synostosis. Premature synostosis commonly involves the sagittal and coronal suture. Lambdoidal sutures are occasionally involved. The craniosynostosis of the sagittal is predominant in boys, while the coronal is more common in girls.^7^ The type of obliterated sutures determines the shape of the cranial vault. The skull shape can vary from brachycephaly (most commonly observed) to scaphocephaly (boat-shaped head), oxycephaly, plagiocephaly, trigonocephaly (triangle-shaped head) or in severe disease cloverleaf skull (kleeblattschädel) like deformity.^11^ In the present case premature closure of the sutures had caused restricted skull growth and lack of space for the growing brain resulted in ‘compensatory’ change in the growth of skull and brain toward frontal region, where the coronal sutures were opened surgically causing frontal bossing creating an elongated narrow scaphocephalic skull.

The facial and oral malformations consist of hypoplastic maxilla and zygoma, pointed nose (psittichorhina/parrot beak-like nose) due to the short and narrow maxilla, narrow high-arched palate, bilateral palatal swellings (pseudocleft) or cleft palate in some patients and crowding of teeth as well as posterior crossbite and reverse overjet with anterior open bite and relative mandibular prognathism.

Approximately one-third of patients with CS suffer from hearing loss due to middle ear deformities and upper airway obstruction occurs due to midfacial hypoplasia and narrow epipharynx.^7^ Optic atrophy is frequently seen and has been reported in 30 to 80% of patients.^12^ Affected individuals exhibit ocular malformations including hypertelorism, proptosis due to the shallow orbits. Mental ability and psychomotor development is generally within normal limits. However, when the premature closure of the cranial suture lines impairs brain development due to increased intracranial pressure it can lead to mental retardation.^7^ The gene for CS could be localized to the FGFR2 at the chromosomal locus 10q 25.3-q26 in more than 50% of cases.^13^ Mutation of the FGFR gene is also responsible for other craniosynostosis, such as Apert's, Pfeiffer's, Jackson-Weiss' and Saethe-Chotzen's syndromes.^14^ Rarely, acanthosis nigricans may coexist with CS in childhood and is caused by mutation in the FGFR3 gene (locus 4p16.3)^15^

Thorough clinical, radiological and genetic analysis is required for early diagnosis of CS. Prenatal diagnostic testing for FGFR gene mutation is an option for couples at risk for having a child with CS. Ultrasonic prenatal diagnosis of exophthalmos might give a clue regarding the developing problems.^16^

The management requires a multidisciplinary approach and the surgical treatment usually begins in the child's first year with cranial decompression. In the presented case early craniectomy of coronal sutures was done at the age of 9 months to relieve increased intracranial pressure caused by premature multiple suture synostosis. An increased intracranial pressure impairs brain development and can lead to mental retardation. Because of the early diagnosis and intervention in this case, no complications were found in our case except for dysmorphic features and the patient presented with normal intelligence. The patient showed speech difficulties because of tongue tie which can be improved surgically by the correction of tongue tie. Skull reshaping may need to be repeated when the child grows and subsequent development of midfacial hypoplasia also needs correction. Procedures for this purpose will include Le Fort III osteotomy or its segmental variants, monobloc frontofacial advancement, or bipartition osteotomy which helps in the cosmetic reconstruction of facial dysmorphisms. The goal is to stage reconstruction to coincide with facial growth patterns and psychosocial development. The prognosis in the case of CS depends on severity of malformation and the patients usually have a normal lifes pan.

In our case, complex treatment plan involving prophylactic and therapeutic approach was formulated for the patient which includes regular mechanical and chemical professional plaque control, with fluoride and chlorhexidine applications to control the intense carioactivity and gingival inflammation. Restoration of existing caries lesions and correction of tongue tie have also been planned to improve speech.

## CONCLUSION

Despite tremendous advances in the establishment of inheritance mode of this condition and its prevention and treatment, it remains a significant cause of morbidity worldwide. Innovations in craniofacial surgery have enabled patients to achieve their full potential by maximizing their opportunities for intellectual growth, physical competence and social acceptance. Dental professionals should have sufficient knowledge of syndromes associated with dysmorphic faces to detect patients who are unaware of their condition, so they may be identified and sent for early investigation and management as required to prevent complications due to late diagnosis.
